# Research on the emotional space design of psychological counseling room from the perspective of embodied interaction

**DOI:** 10.3389/fpsyg.2026.1950761

**Published:** 2026-09-23

**Authors:** Wenjing Bai, Yuchun Leng, Guanguan Wei, Yang Li

**Affiliations:** 1 School of Arts, Chongqing Technology and Business University, Chongqing, China; 2 School of Arts and Media, Chongqing Metropolitan College of Science and Technology, Chongqing, China

**Keywords:** counseling room, design practice, design strategy, embodied interaction, emotional space

## Abstract

The design of a supportive counseling room is closely related to the effectiveness of psychological counseling, and embodied interaction is an important way for clients to obtain counseling experiences. In order to guide the environment design of counseling room applying embodied cognition theory, this paper analyzes the clients’ embodied interaction process in counseling room, interprets the spatial environmental design elements of counseling rooms from the emotional dimension, clarifies the design elements of psychological field based on the Gestalt psychological effect, analyzes the relationship of psychological theoretical findings, design goals and design content, and proposes emotional space design strategies for counseling rooms from the aspects of emotion arousal, emotion experience, emotion identification, and emotion co-construction. These strategies are applied in design practice, providing a reference for the application of environment-related psychological research and the environmental design practice of psychological counseling rooms.

## 1 Introduction

Embodied cognition is a significant theory in cognitive science at the end of the 20th century, positing that cognition arises from the dynamic interaction between the body and the environment. Barker (1968) proposed the theory of behavior setting, holding that the material characteristics of the environment support certain fixed patterns of behavior. Altman (1976) further pointed out that privacy is a dynamic social process influenced jointly by individual, environment and cultural factors, and is meanwhile regulated through various behavioral mechanisms. These researches provided a direct theoretical and fundamental framework for the further study and application of embodied cognition. Lakoff and Johnson (1999) systematically demonstrated the dependence of cognition on the body, arguing that abstract thinking is rooted in bodily experiences and sensorimotor systems. Barsalou (1999) proposed the theory of Perceptual Symbol System, elucidating that cognition is not a disembodied calculation, but rather a simulation process deeply integrated with the sensory and motor systems, which provided a high-level functional account of how the brain could implement a conceptual system using sensor-motor mechanism. On that basis, Barsalou (2008) further demonstrated the body-dependency of concept processing from the perspective of psychological experiments, becoming an important component of embodied cognition theory.

The embodied cognition theory emphasizes that cognition stems from embodied practice and contextual perception, laying the foundation for human-centered spatial environment design. Space is not only a physically existing entity, but also a compression and symbol of social relations, with the triple attributes of materiality, spirituality and sociality (Lefebver, 1974). The study of environmental psychology has also confirmed that the spatial environment has an significant impact on people’s emotions, cognition, and behavior. Holl et al. (2006) declared that architectural design evokes individual’s experience through light, materials, space and scale. Juhani (2012) criticized the visual centrism of architectural experience and emphasized on the core position of multi-sensory experiences and “the presence of the body”. In a recent study, Barth and Blazejewski (2023) explored how the open office environment is restructured through embodied practices in actual use, emphasizing that spatial practices are the core for spatial experiences. In the highly sensitive and introspective field of psychological counseling, the role of the spatial environment on human’s emotion may be further amplified. A counseling room is not merely a place providing basic functions, but also a non-verbal communication medium, continuously conveying information about safety, acceptance, professionalism and care to clients. A well-designed counseling room can become a “safe base” that promotes healing, while an improperly designed space may invisibly increase clients’ defensiveness and anxiety, becoming an obstacle to establishing a “therapeutic alliance.”

Therefore, as a therapeutic container, the significance of the spatial environment of counseling room has been increasingly recognized by academic researchers, and designers as well. It is believed that through environmental elements such as light, color, sound, material, smell and spatial layout, a unique “spirit of place” is shaped in the counseling room. From the moment clients step into the counseling room, they begin to receive and interpret signals from the environment. These initial impressions often influence their perception of the counselor, their expectations of the counseling process, and their willingness to open up and explore deeply, thereby subtly supporting or hindering the achievement of the goals of psychological counseling. Thus, how the embodied cognition theory can guide the design of counseling room has become a focusing topic in the field of environment design studies in recent years.

The core of psychological counseling lies in interpersonal interaction, that is, the unique relationship established between the client and the counselor, which always takes place within the specific physical space of the counseling room, and is frequently affected by the client’s individual emotion. Relevant researches generally hold that emotion is a complex psychological phenomenon, a psychological activity process composed of multiple components, with a multi-dimensional structure and multi-level integration, and interacting with cognition due to the organism’s survival adaptation and interpersonal communication (Zhao, 1994). Some studies also hold that emotion is an induced response, and the spatial environment is the external attribution for inducing emotions (Xia, 2019). Miwa and Hanyu (2006) studied the impact of different indoor lighting levels on the emotions of counseling clients through contrast experiments, revealing the psychological effects of counseling room lighting. Pearson and Wilson (2012) studied the impact of color design on clients’ emotions and the effectiveness of psychological counseling, revealing that children and young people usually associate light colors with positive emotions, while anxious adults tend to prefer white or low-saturation office environments. Kim (2010) found that the arrangement of counselors and the placement of furniture also affect clients’ self-disclosure. Sandra and Kathy (2023) argued that acoustic privacy is also important for the environmental design of counseling rooms, as noise can affect clients’ levels of fear and anxiety, thereby disrupting the counseling environment. Above mentioned studies show that environmental factors including lighting, color, furniture arrangement and noises can interact with clients and arouse their different emotional responses. These responses further affect clients’ perceived safety and willingness to self-disclose, and in turn, influence the therapeutic alliance relationship between clients and counselors.

Under the influence of embodied cognition research, the field of design has also begun to incorporate the relationship between the body and the environment into its research scope, with a focus on how humans establish an interactive relationship with the environment through their bodies. Dourish (2001) initially proposed the theoretical framework of embodied interaction, advocating that interaction design should focus on the physical practices and social contexts of users in the real world, rather than merely concentrating on abstract rational cognition or screen interfaces. Lindgaard and Wesselius (2017) took design thinking as a way of cognition to explain the intuition, sensibility and experience in the design process based on metaphor and embodied cognition theory. Jerry (2019) further argued that design thinking is an “embodied” experience process, and design activity is a process of the synergy between the brain, the body and the environment. Klippel et al. (2019) studied the supporting technique and immersive experiences of embodied cognition through experiments of 3 virtual field trips, and proved an overwhelmingly positive response of students to virtual field trips with significantly higher-valued learning experience and enjoyment and a significant improvement in the participant’s spatial situation model when introduced the pseudo-aerial imagery. In the context of escalating global public health and individual mental health challenges, embodied interaction offers new perspective for health design, environmental design, and healing space design. Liu et al. (2024) discussed the embodied interaction, design practice and the value of environmental medium in art therapy process, and analyzed the functional model of environmental media in healing spaces through design cases. However, the embodied interaction of the clients in the counseling room is not covered yet, and the relevant research findings in embodied cognition have rarely been applied in the design of counseling rooms so far. The main purpose of this paper is to establish a basic framework of the client’s embodied interaction in counseling room, and to guide the design of the counseling room based on these theoretical findings. The research process is shown in Figure 1.

**Figure 1:**
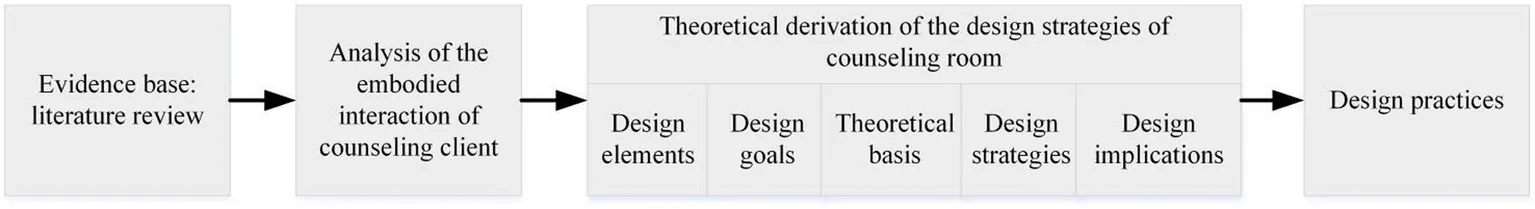
The research process of this paper.

## 2 Analysis of the clients’ embodied interaction

The lived world as a setting or background brings interaction design back to the realm of daily life, and the essence of embodied interaction is to design based on daily skills, with the core of interactivity, embodiment and metaphor (Lu and Liu, 2013). Shan and Mei (2021) studied the interactive behaviors of children and different space environments through investigation and behavior observation, and derived the spatial design principles adapted to children’s physical activities and cognitive development based on the theory of embodied cognition, which provided some theoretical basis and design guidance for the healthy design of children’s playing space. Qin (2024) studied the relationship between soundscape design and body sense in electronic sports, and pointed out that the soundscape promotes the construction of a holistic field and bodily sensation and forms a psychological connection of rhythm isomorphism with the competitors. In this way, soundscape design facilitates the completion of the competitor’s gestalt embodied cognition. By employing research methods such as grounded theory, fuzzy set qualitative comparative analysis and field investigation, Su et al. (2024) explored the constituent elements and scientific configuration paths of the embodied interaction space in libraries, and proposed the strategies for libraries spatial design. It can be concluded that embodied cognition focuses on the relationship between the body and space, considering how humans acquire the understandings of space through their bodies, while embodied interaction is studied as a form of interaction between humans and the environment, where the body itself serves as the interface, providing the fundamental of spatial design and environment design.

During counseling process, the spatial environment does not directly “cure” clients, but functions through mediating variables such as perceived safety, self-disclosure, and the therapeutic alliance. As shown in Figure 2, in a typical psychological counseling process, clients actively engage in an embodied connection with the environmental media of the counseling room. Through embodied contact and timely feedback, they receive the metaphorical expressions (metaphor hint) from both the counselor and the environmental media (including the five senses, kinesthetic sense, and sense of power), thereby enhancing their perceived safety. They release repressed subconsciousness and relieve intense inner emotions in a symbolic, non-verbal manner, which, together with verbal expression, conversation, and questioning, forms a holistic perception (integration gestalt) and promotes self-disclosure. On the other hand, in addition to the verbal communication with the counselor, clients also integrate the counselor’s behavior and environment in an implicit manner (tacit perception), forming a therapeutic alliance. This makes the environmental media of the counseling room an active auxiliary to the counselor’s work. Through the embodied interactive experience of language and environmental media, clients unconsciously perceive and face their emotions, enhance self-awareness, and control self-expression, thereby achieving psychological healing.

**Figure 2:**
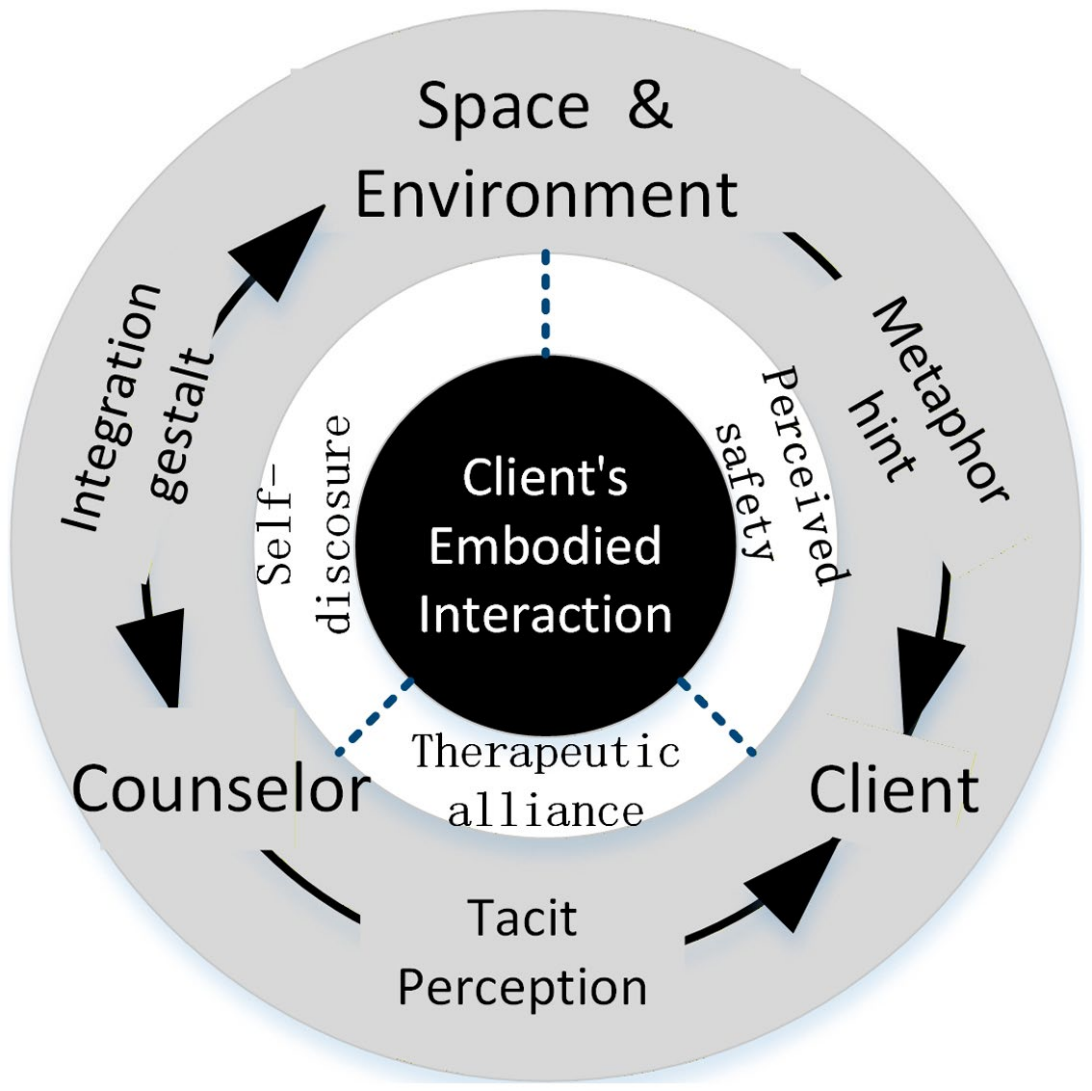
The embodied interaction process of counseling clients.

The theory of embodied interaction emphasizes that cognition is the result of the dynamic coupling of the body, the environment, and the mind (Zhang and Song, 2026). A client’s experience in a counseling room is not a passive reception of environmental stimuli, but a dynamic process of continuous interaction with the spatial environment through bodily perception and behavioral actions. When a client steps into the counseling room, his body’s sensory systems—vision for color recognition, skin’s tactile feedback to temperature and materials, and ears’ capture of echo and sound insulation quality—immediately couple with the environment, forming a pre-reflective bodily perception. These micro-adjustments at the bodily level often precede conscious language, constituting the “pre-linguistic” foundation of the counselor-client interaction. Clients do not think first and then act; instead, they perceive in action and adjust their cognition in perception. When a counselor guides a client to move from the sunlit window to an indoor green plant or art corner during a session, the physical movement itself participates in the transformation of the emotional state. This ternary coupling of body-environment-mind indicates that the spatial environment design of a counseling room is not merely “room decoration,” but an intentional intervention in the client’s entire cognitive ecosystem. By regulating the physical structure and interactive interface of the space, it provides an embedded support platform at the bodily level for psychological healing.

In addition, Narrative Therapy, a typical approach in postmodern psychological counseling, holds that every individual has sufficient positive experiences and the initiative to overcome obstacles (Wang and Guo, 2022). Therefore, the space environment of a counseling room that evokes positive emotional experiences can help clients discover and confront their own strengths, enhance their awareness of self-disclosure, and promote the formation of the therapeutic alliance, thereby improving the practical effectiveness of psychological counseling. In practice, art healing primarily achieves the goals of emotional comfort, anxiety relief, and disease treatment through non-verbal artistic creation, with emotional cognition at the core of its interaction (Wang and Wang, 2025). In contrast, psychological counseling mainly achieves psychological assistance and obstacle elimination through verbal interventions such as storytelling, questioning, and discussion. Although the main methods to achieve their goals differ, for those seeking healing or counseling clients, the embodied interaction process in the environmental media is similar. Both involve constructing, manipulating, and conveying meaning through interaction with the environment and others—embodied interaction is precisely the process of meaning creation, use, and dissemination during participatory interaction between people and the environment, others, or other objects (Dourish, 2001). Essentially, psychological counseling activities involve clients completing the construction of meanings such as emotional relief, psychological guidance, and obstacle elimination through embodied interaction in the spatial environment of the counseling room.

## 3 The emotional space design strategies of counseling room

### 3.1 The design elements of counseling room

In order to discuss the role of light environment design in the narrative of urban cultural tourism spaces, Tan et al. (2026) proposed the concept of “emotional map” to analyze the narrative logic of “light induces emotion - emotion drives path selection - path constructs spatial expression” in the design of historical blocks. Similarly, this paper puts forward the concept of “emotional space.” On the one hand, it refers to the inducing and promoting effect of the counseling room’s spatial environment on clients’ emotions. On the other hand, it emphasizes that “the space itself has emotions”, which can directly envelop and immerse the clients in it, forming an emotional space that precedes language and cognition. Once clients enter it, they integrate with it through embodied interaction.

The embodied interactive experience originates from all sensory channels of the human body. Environmental media such as the space, shape, light, color, sound, smell, and touch of the counseling room all influence clients’ subjective cognition and psychological feelings, triggering different emotional responses through multi-dimensional and stereoscopic means. Among these, tacit understanding plays a crucial role in the formation of clients’ embodied interactive experiences (Hu and Hu, 2019). The way of tacit comprehension is usually contextual, invisible, indescribable, and holistic. Gestalt psychology holds that people’s perception of things is not merely a simple accumulation of individual elements, but rather they perceive these elements as a whole (Wang et al., 2009). Similarly, artistic creation achieves a state of perfection, self-sufficiency, and balance through the combination of various elements such as shape, color, and materials, which is the most perfect state of the work’s structure, namely “Gestalt” (Hu and Hu, 2019). In other words, clients’ embodied interactive experiences are gestalt and holistic. They actively understand the metaphors of environmental media through embodied perception and complete the construction of meaning in combination with verbal interaction with the counselor. On the other hand, clients’ emotions are triggered by the stimulation of environmental media. As a carrier of emotions, the space shapes the environmental atmosphere through various factors such as its layout, circulation, color, lighting, furniture, and decorations. The overall stimulation is greater than the sum of the individual environmental elements, which is the Gestalt psychological effect of the spatial environment of the psychological counseling room.

Therefore, the emotional space design of a counseling room should be “Gestalt” that progresses from localized emotional relief to overall emotional co-construction (shown in Figure 3). It is necessary to consider both individual design elements such as space/route, walls/shape, floor/ceiling, furniture/lights, and ornaments/decorations, as well as the psychological hints and emotional stimuli they bring to clients, such as a sense of security, privacy, trust, and empathy. Additionally, it should take into account the design elements of the psychological field, including indoor odor, sound, light perception, tactile sensation, and food. By comprehensively utilizing the five senses, sense of movement, sense of strength, and individual spatial cognition, clients can obtain a positive Gestalt psychological effect, thereby reaching the goal of positive emotional co-construction (that is, the meaning construction of clients’ embodied interaction).

**Figure 3:**
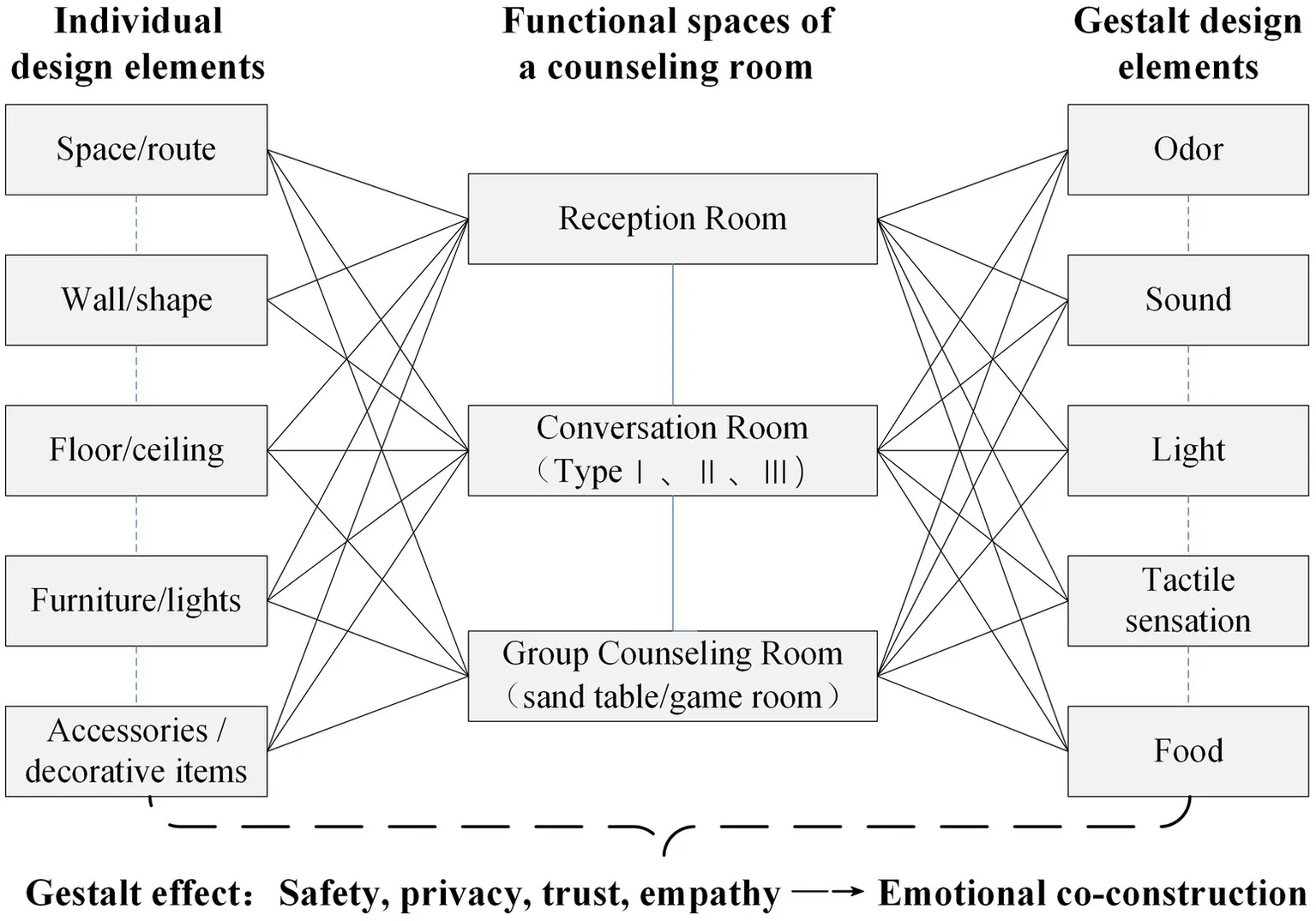
The spatial design elements of counseling room.

### 3.2 The theoretical derivation of the design strategies of counseling room

In landscape color design, Shen et al. (2026) reviewed and synthesized the psychological and behavioral impact of landscape color through a four-dimensional analytical framework encompassing time, space, visual stimulus, and population heterogeneity, and suggested that landscape color design should adopt differentiated, evidence-based strategies tailored to exposure duration, spatial context, and target user group. The basis of the aforementioned four-dimensional framework lies in the analysis of the action mechanism by which the landscape color affect humans: perceptual interpretation, cognitive processing and emotional regulation. Similarly, the analytical framework of the environment design strategy of counseling room is established by the different design goals: emotion arousal, emotion experience, emotional identification and emotion co-construction, as shown in Table 1, coinciding with the different phases of the emotional construction that the clients undergo in counseling room.

**Table 1:** The analytical framework of the design strategies.

Design goal	Theoretical basis	Design strategies	Design implications
Emotion arousal	Human’s emotional responses of the environmental stimulus.	Adaptive design (Design for adaptability)	Design the individual spatial elements and visual stimulus, such as color, light, sound, material, furniture arrangement and others according to the emotional effects of those environmental media.
Emotion experience	Embodied interaction and embodied cognition, stress recovery theory.	Consistent design (Design for consistency)	Design the spatial narrative logic and the route of client’s embodied interaction, such as the arrangement of furniture, the counseling procedure, the path planning, interior ornament, vegetation and plants etc.
Emotion identification	Place attachment theory, socio-cultural meaning, transference and empathy.	Differentiated design (Design for differentiation)	Design “meaningful” interior environmental atmospheres that enable clients to achieve emotional identification through the mechanisms of apperception, transference and empathy. For instance, distinct interior decoration styles and ornamental objects may be employed to correspond to the particular affective states of different clients.
Emotion co-construction	Tacit perception, gestalt psychology.	Holistic design (Design for integration)	The holistic influence of the Gestalt design elements enumerated in Figure 2 must be taken into consideration in design, in order to co-construct the positive affect with the clients through the metaphors embedded in the environmental design.

Table 1 synthesizes the literature research findings in Introduction and declares the theoretical basis of the design strategies. For emotion arousal purpose, the main design work is to deal with the interior elements such as the lighting, material, color, noise and so on, according to clients’ timely responses up them. The affective influence of light, material, color, noise has been extensively studied (Miwa and Hanyu, 2006; Pearson and Wilson, 2012; Sandra and Kathy, 2023), and a resent research declares that the test subjects in the brightly lit and quiet room walk more slowly and are more likely to stay in the room, and the emotional design of interior environment mainly refers to the design of color, material, sound, light and space arrangement (Wang et al., 2023).

For emotion experience creating, researchers and designers have long focused their attention on the study of users’ embodied cognition and embodied interaction (Jerry, 2019; Lu and Liu, 2013; Shan and Mei, 2021). User experience (UX) is one one of the key focuses of current design research. In 2010, the International Organization for Standardization defined user experience as the perceptions and responses of a user that result from the use or anticipated use of a system, product, or service (International Organization for Standardization, 2010). Regarding user experience design, Schaffer (2009) proposed the concept of “beyond usability,” linking user experience with sustained behavior, arguing that UX design should not only support what users can do, but should also build trust by enhancing persuasiveness and evoking emotions, thereby promoting users’ sustained engagement and behavioral change. This offers important implications for the spatial environment design of counseling rooms: on the basis of ensuring the clients’ basic needs for functionality, safety, and communication, a consistent emotional experience should be created for clients through narrative spatial layout, behavioral circulation routes, interior decoration, and five-senses design, thereby facilitating the formation of a positive experience. In addition, Stress Recovery Theory also provides a theoretical foundation for the emotional experience design of consultation rooms. This theory holds that naturalistic environmental features can help reduce stress responses and allow individuals to gradually recover from a state of high arousal (Ulrich, 1984; Ulrich et al., 1991). Therefore, in the spatial environment design of counseling rooms, attention should be paid to the use of natural elements, such as greenery and designs with natural forms.

For emotion identification, place attachment theory (PAT) provides the fundamental theoretical support. Place attachment theory focuses on the emotional, cognitive, and behavioral bonds formed between individuals and specific environments, as well as the impact of these bonds on psychological adaptation and social behavior (Altman and Low, 1992). Places with personal significance can provide emotional regulation resources and support a stable sense of self (Korpela et al., 2001). Therefore, the spatial environment design of counseling rooms should help clients identify unique meanings within the space that align with their individual needs, and adopt differentiated and diverse interior “atmosphere” designs based on the emotional states of different clients.

For emotion co-construction, the holistic psychological effect must be taken in account in the spatial environment design of counseling rooms, with the key points of the Gestalt design elements shown in Figure 2. Multiple sensorial channels should be integrated to create a holistic immersive emotional experience, thereby enhancing the effects of emotional appeal and co-construction in counseling rooms. In terms of design details, more attention should be paid to the unity of spatial design elements - such as color, lighting, materials, furniture, and decoration - which should form a unified “Gestalt,” creating a harmonious, safe, and stable overall atmosphere and eliminating fragmented perceptions. The spatial layout should also be used to imply privacy and equality in conversation, guiding visitors toward a psychological “closure” of the space, so that the space becomes an invisible medium for promoting self-discosure in counseling process.

### 3.3 The emotional space design strategies of counseling room

The specific design strategies are summarized based on Table 1. Firstly, the adaptive environment design strategy focuses on the metaphorical implications of environmental media to achieve the goal of arousing positive emotions in clients. The environment design of a counseling room should firstly consider the basic needs of users, including functional needs, physiological needs, safety needs, identity and respect needs, and self-actualization needs. The adaptive design strategy emphasizes on the adaptation between the general and the special, the mutual promotion of safety and trust, the balance between public and private spaces, and the complementarity between professionalism and relaxation. Through adaptive emotional space design, a stable, warm, safe, and supportive environmental atmosphere is created. Through designs of color, light, sound, touch, and other individual elements, psychological hints of peace and tranquility are conveyed to evoke positive emotional responses in clients, enabling them to engage in self-exploration and achieve self-growth.

Secondly, the consistent environment design strategy focuses on spatial narrative logic and embodied interaction route design, aiming to create positive emotional experiences through an imperceptible and gradual process. The experience clients gain in a counseling room is continuous, non-verbal, and accumulative. From the geographical location of the counseling room, the environmental atmosphere of the reception area, the behavioral circulation indoors to the arrangement of furniture and ornaments, clients obtain an overall experience through every touchpoint throughout the entire counseling and communication process. Consequently, the reception procedure, spatial layout, partitions, transitions, connections, as well as indoor spatial form, color, light, and materials of the psychological counseling room should all meet the requirements of continuity, complementarity, and coordination. These elements should advance in a progressive and layered manner, jointly providing clients with a comfortable, friendly, and supportive healing environment.

Thirdly, the differentiated environment design strategy focuses on shaping a targeted, “meaningful,” symbolic and non-verbal environmental atmosphere through the mechanisms of apperception, transference and empathy,with the aim of facilitating communicative interaction between the client and the counselor and thereby achieving emotional identification. Clients typically confront a diverse range of psychological distress and exhibit pronounced individual differences in personality traits, emotional expression, cognitive ability, learning capacity, and empathic ability. Accordingly, the counseling room should, on the premise of adaptive design, provide flexible, embodied participation solutions for different clients. On the one hand, the distinction between individual counseling and group counseling must be taken into account, and the overall spatial planning of the counseling room should be flexibly arranged to strike an appropriate balance between spatial “privacy” and “openness,” while avoiding overly open spatial layouts that may induce anxiety and tension. On the other hand, the unique needs of individuals at different emotional stages should also be considered: furnishings and decorations, audiovisual materials and music, and refreshments and aromatherapy should be flexibly configured in accordance with both individual and group needs, so as to provide distinct spatial environments for clients presenting with different manifestations of psychological discomfort, thereby achieving the goal of emotional identification.

Fourthly, the holistic environment design strategy focus on the Gestalt design elements enumerated in Figure 2, aiming to co-construct the positive affect with the clients through the metaphors embedded in the environment design, along with the clients’ tacitly apprehension of counselor’s body language and facial expressions. Both the spatial environment of the counseling room and the counselor’s kinesthetic-verbal interventions exert a joint influence on the client through metaphorical and tacit means, thereby shaping the client’s holistic impression of the counseling experience. In particular, as discovered in past consulting experiences, the client tends to infer the counselor’s emotional preferences and attitudinal dispositions from cues such as body movements, facial expressions, and vocal intonation, and subsequently forms a subjective judgment as to whether the counselor demonstrates empathy toward him or her. An appropriately designed counseling environment should thus operate in concordance with the counselor, facilitating the co-construction of positive affect with the client. Although targeted design interventions—including color, form, lighting, and texture—may alleviate the client’s emotional distress at the level of emotional relief, from the perspective of Gestalt psychological effects, the spatial environmental design of the counseling room should shift from localized emotional relief toward holistic affective co-construction. This entails systematically planning the local visual design elements alongside the Gestalt design elements at the level of the psychological field (as illustrated in Figure 2), so as to comprehensively evoke the client’s positive emotional resonance through the five senses and the perception of personal space, thereby achieving the objectives of affective co-construction and psychological counseling.

In conclusion, the design strategies proposed above are consistent with the progressive logic by which clients’ emotions advance from arousal, through experience and identification, to co-construction, and they likewise constitute a synthesis of design requirements examined from multiple perspectives on the environment design of counseling room. On the whole, the environment design of a counseling room oriented toward clients’ embodied interactive experience should be narrated from the perspective of emotional space construction, creating an immersive Gestalt space for emotional co-construction and resonance among clients through connected spaces, transitional scenarios, vibrant colors, flowing music, drifting light and shadow, and faintly perceptible scents. Such a space serves the positive functions of emotional expression, cognitive regulation, emotional contagion, and psychological cultivation, thereby collaborating with the professional support provided by the counselor to achieve the goal of healing.

## 4 The design practice of a college counseling room

Studies on how environment design induce emotions show that the clarity and coherence of boundaries and paths, as well as the definiteness of spatial shapes and outlines, are significantly positively correlated with the arousal of relaxation emotions (Han and Zhang, 2022). The environment design of college counseling room should, first and foremost, clarify the spatial layout of its interior functional areas, clearly delineate the boundaries of each functional zone, and plan behavioral circulation routes in accordance with the needs of different client groups. Guided by the aforementioned adaptive, differentiated, and consistent design strategies for the counseling rooms, college counseling room may be holistically divided into such functional units as the reception and administrative zone, the rest and waiting zone, the multifunctional group counseling room, and the individual counseling room. Distinct circulation routes should then be designed separately for group clients and individual clients, so as to ensure the continuity of these routes while accommodating the relative privacy of different clients.

As illustrated in Figure 4 (designed by the authors), the boundary of the individual counseling room is designed with an arc-shaped contour, characterized by clear boundaries, simple forms, and bright, warm colors, which serve to evoke a relaxed emotional state in clients. Meanwhile, in accordance with the varying needs of client students, the individual counseling room may be subdivided into three functional zones: the entrance & privacy-protecting partition area, the sofa counseling area, and the hypnotic therapy area. These three areas are separated from one another by partitions and furniture, and their spatial dimensions correspond to the behavioral scale of the human body, thereby providing clients with a sense of psychological safety without inducing feelings of oppression.

**Figure 4:**
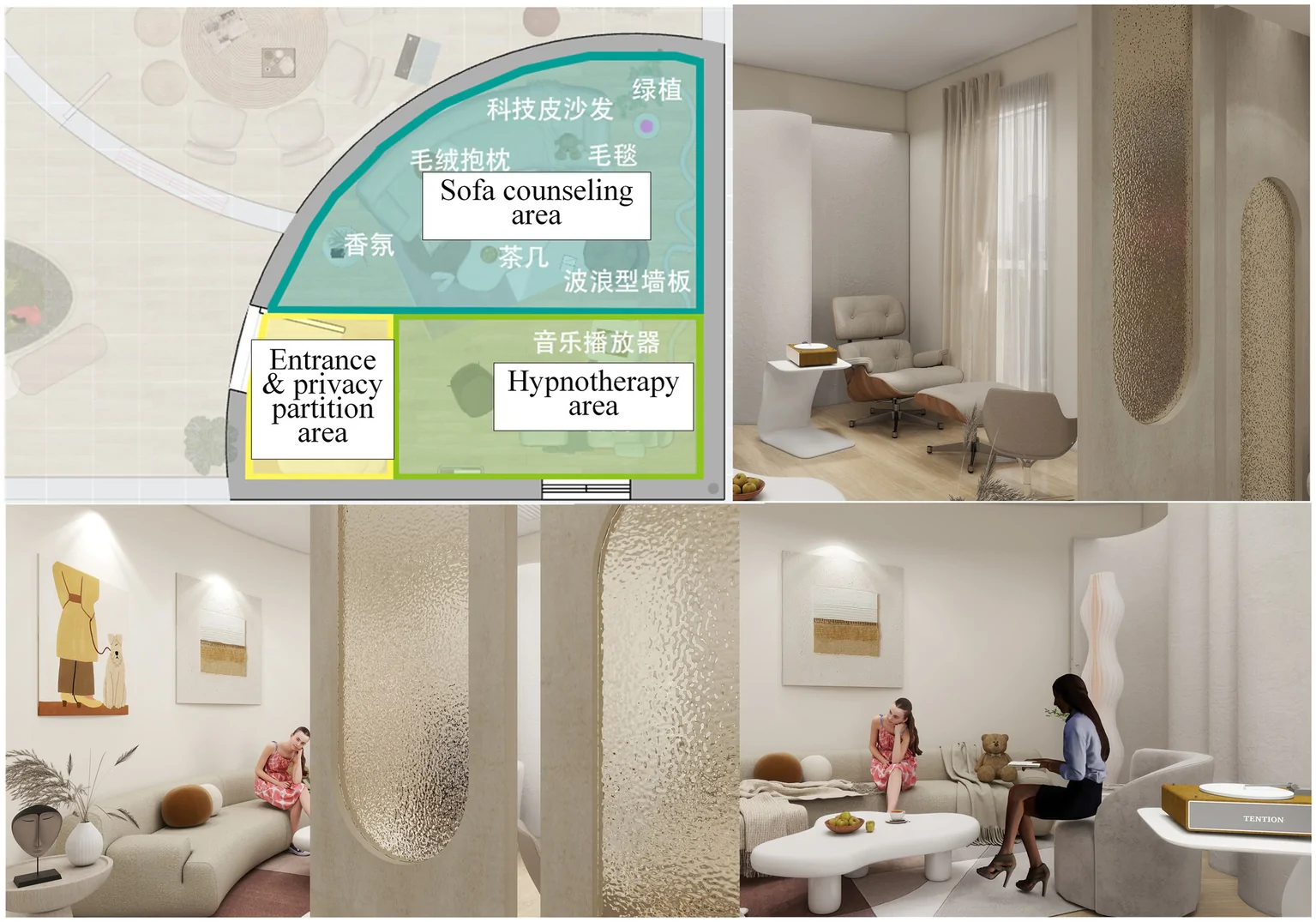
The design reference of individual counseling room.

The entrance & privacy-protecting partition area employs split-type opaque frosted glass, so as to avoid the sense of oppression and coercion that an overly large partition might engender, while fostering a safe and private environmental atmosphere. In the sofa counseling area, the furniture consists of off-white fabric sofas, whose surfaces exhibit a soft texture and warm, supple tactile qualities; the coffee table is designed with a sturdy, full-bodied form, conveying a formal language of safety and reliability. At the same time, the embodied cognition effects of clients are given full consideration: through organically shaped lighting fixtures, fully proportioned vases, and brightly colored, texturally rich hanging ornaments and decorative objects, a collective psychological suggestion of tranquility, confidentiality, reliability, professional trustworthiness, and comfort is established. Furthermore, the hypnotic therapy area is concurrently designed as a diversified artistic creation space, enabling clients to engage in painting, doodling, or other forms of artistic expression, thereby integrating hypnotherapy with art therapy in the treatment approach.

As illustrated in Figure 5 (designed by the authors), the group counseling room is designed as an irregular space formed by a combination of two arc-shaped sections. Although it is nested with the individual counseling room in terms of layout, each has its own main circulation path and entrance to ensure relative independence. According to the main types of group psychological counseling activities, the group counseling room is divided into three zones: a sand-play area, an adaptable group counseling area, and a stress-relief area. Studies have shown that analogous colors are most effective in evoking a sense of calm and tranquility, and changes in lightness and chroma have a greater impact on one’s emotional responses than changes in hue. In particular, changes in lightness have a significant influence on emotions (Yu et al., 2014). Therefore, the main color of the group counseling room is consistent with the main color scheme of the individual counseling room, with almost no changes in lightness and chroma. Large areas of off-white walls and furniture are used, together with warm-toned flooring, to create a relaxed and pleasant environmental atmosphere.

**Figure 5:**
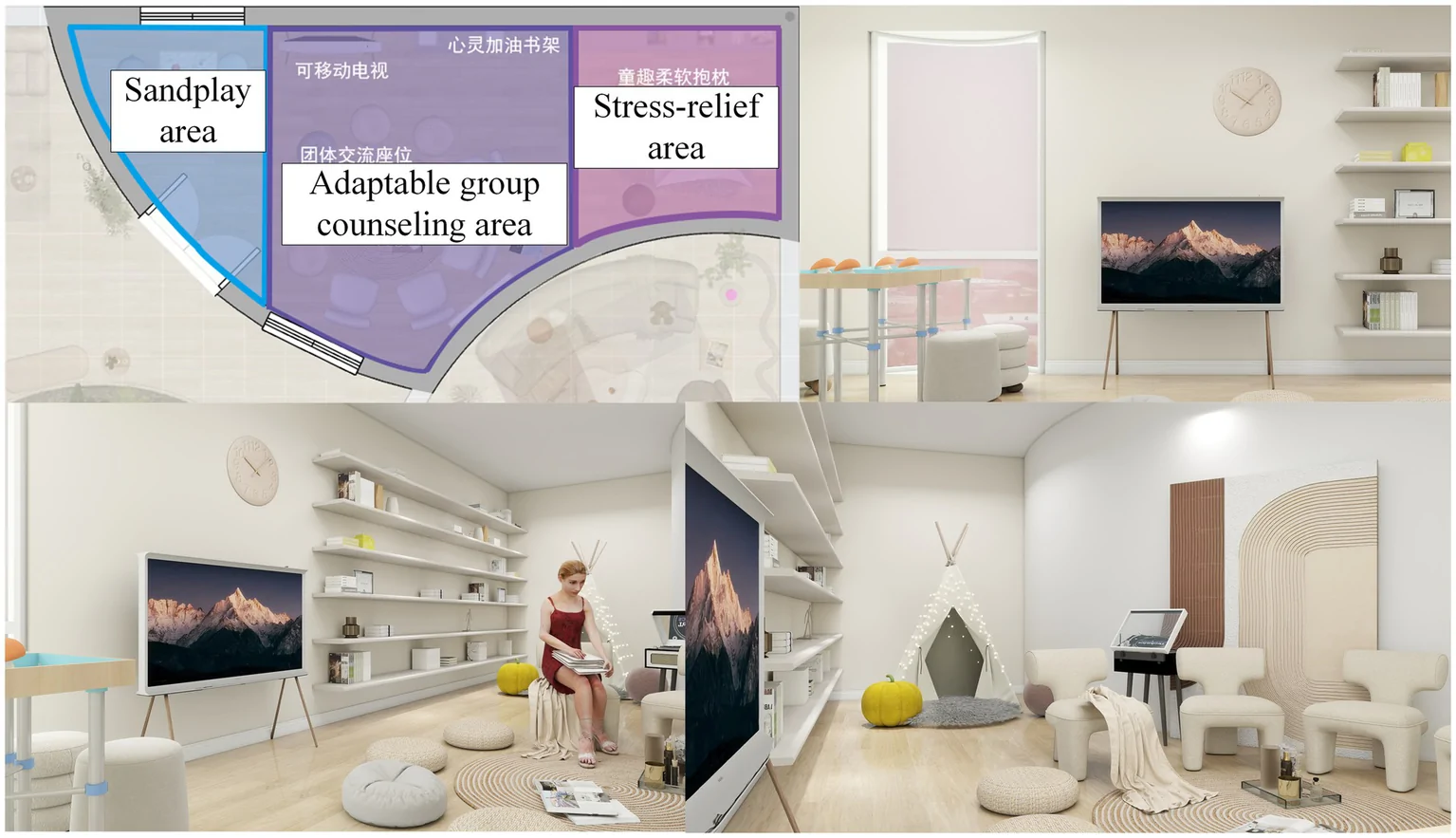
The design reference of group counseling room.

The “Psychological Encouragement Bookshelf” has a concise design, conveying a psychological implication of rational restraint. The cushions, chairs, stools, and other furniture are designed with rounded and endearing forms, rendered in a neutral gray-white color scheme and upholstered in linen characterized by a soft texture, fine grain, and warm tactile quality. Through both visual and tactile channels, these elements collectively prompt students’ clients to develop a positive psychological field effect characterized by calmness and serenity, relaxation and pleasure, rational acuity, and restrained modesty.

From the perspective of embodied interaction, in addition to the above-mentioned localized emotional relief designs in terms of vision and touch, comprehensive application of Gestalt design elements has also been carried out in aspects such as hearing, smell, and taste, aiming to achieve the goal of emotional arousal and co-construction between the spatial environment and clients. In the individual counseling room, the record player, and in the group counseling room, the mobile TV and phonograph can play different types of audio and video according to different counseling contents and group counseling themes, assisting in evoking positive emotions in clients from the auditory aspect. Vases, aromatherapy, snacks, fruits, etc. provide moderate stimulation of smell and taste. Moreover, pumpkin-shaped cushions and bread-shaped desktop ornaments provide clients with a space for association and imagination. The complementary stimulation of smell and taste enhances the cohesion and appeal of the emotional space in the counseling room. Pleasant aromatherapy and themed taste experiences can enhance the sense of ritual, integration, and wholeness of the space, helping clients obtain a holistic embodied cognition and emotional experience.

Furthermore, previous studies have demonstrated that the experiential efficacy of VR-based art therapy is significantly more pronounced than that of traditional approaches (Peng et al., 2025). Accordingly, future design should actively incorporate emerging media technologies—such as virtual reality, intelligent interaction, digital media, and biofeedback—to reconstruct the psychological field of the counseling room. This endeavor should not only ensure the professional rigor of psychological support, but also enhance the emotional inclusiveness of the spatial environment and deepen the immersive quality of clients’ embodied interaction. Three-dimensional displays, head-mounted devices, and intelligent interactive projection systems can be integrated with the physical environment to construct an augmented reality environment combining virtual and real elements. Such a configuration not only evokes positive emotional responses through immersive multisensory stimulation, thereby helping clients establish embodied cognitive connections, but also captures clients’ subtle behavioral and emotional data through non-verbal means, thereby providing counselors with objective auxiliary diagnostic evidence.

## 5 Conclusion

Drawing upon existing findings in environmental psychology, as well as analyses of clients’ embodied interaction, this study proposes spatial environment design strategies for the counseling room and completes the corresponding design practices. The emotional space design of a counseling room should be a Gestalt that progresses from the localized to the holistic. Since clients communicate with the spatial environment through embodied interaction, the environment design of the counseling room should pay particular attention to the treatment of such design elements as audition, olfaction, and gustation. These Gestalt design elements can evoke clients’ positive emotional responses, enhance their self-awareness, and improve their capacity to tacitly apprehend the counselor’s empathic responses, thereby facilitating inner integration and personal growth within the process of emotional co-construction.

Nevertheless, a notable limitation of the present study lies in the absence of empirical testing and evaluation of the designed counseling environment. Future research will employ interviews and questionnaire surveys to conduct design evaluations of the counseling room proposed herein, and will undertake quantitative investigations into the design efficacy of the spatial environment, the embodied interaction pathways of student clients, and the mechanisms of their embodied cognition. On this basis, service design related to psychological counseling will be further developed—through in-depth exploration of clients’ latent needs, optimization of service processes, and innovation of service touchpoint design—so as to continuously enhance the mental health service experience by leveraging mobile internet and emerging media interaction technologies.
